# Book Reviews

**Published:** 1918-06

**Authors:** 


### BOOK REVIEWS

**Books mentioned may be inspected at and ordered through this office. So far as possible, books received in any month will be reviewed in the issue of the second month following. Pamphlets, quarterly and similar periodicals, reports, transactions, etc., will, as a rule, merely be mentioned.**

---

**A Few Minutes With the Urine.** By J. Henry Dowd, M. D., Genito-urinary Surgeon, Buffalo Hospital, Sisters of Charity, Mercy and Contagious Hospital, Consulting G-U Surgeon, Emergency Hospital, Lieut. (special service) and Lecturer on Insanity, Buffalo Police Department—a 40 page booklet.

In this booklet, which has been written by request, the author says, "If this booklet was for the laboratory man, or the genito-urinary specialist, more would have been written. It is intended for the general practitioner—the man who has not expensive apparatus for technical examination; has not the time did he possess them, and possibly has not a class of be honest in his opinion as far as time, knowledge and circumstances will permit." After mentioning the diseases and patients that can pay for such time given, still he wants to conditions ascertainable from a few minutes spent with the urine, he fully describes the methods of procedure in arriving at a conclusion, also giving the best treatment for the same. The urine is one of the greatest aids in diagnosis we have; Dr. Dowd has covered all the essential points in a most practical and scientific manner. A copy of this little work should be in the hands of every general practitioner of medicine; it is of inestimable value in diagnosis.

The Richardson Co., 334 Franklin Street, for whom this booklet was prepared, will send any physician a copy on request.

---

**The Elements of the Science of Nutrition.** By Graham Lusk, Ph.D., Sc.D., F.R.S., (Edin.), Professor of Physiology at Cornell Medical School, New York. Third edition. Reset. Octavo of 641 pages, illustrated. Philadelphia and London: W. B. Saunders Company, 1917. Cloth $4.50 net.

The author has brought together an immense amount of scientific data, well arranged and presented in logical and attractive form. The first chapter, reviewing the progress of the science of nutrition down to modern times, furnishes the key to the whole book. The next chapter describes the Atwater-Rose respiration calorimeter. After discussing starvation, and the regulation of temperature, about 150 pages are given to the influence of protein food, fat and carbohydrate respectively. The influence of mechanical work on metabolism is presented in a very interesting chapter which prepares the student for the consideration of "a normal diet," and the nine succeeding chapters dealing with diet and metabolism under various conditions of health and disease. The last chapter, on "food economics," is particularly valuable because of its review of the food situation in Germany as affected by the war. The needs of our own country are pointed out, and several pages are devoted to the cost of our foods compared with their nutritive value. The appendix contains the statistical tables of Atwater giving the composition of ordinary food materials. We know of no other book presenting so extensive an array of facts on nutrition in a form that can be readily utilized by the physician and dietitian.

---

**Transactions of the Thirty-ninth Annual Meeting** of the American Laryngological Association held at Atlantic City, N. J., May 28, 29 and 30th, 1917. Dr. Harmon Smith, 44 West 49th St., New York City, Secretary.

---

**Progressive Medicine.** A quarterly digest of advances, discoveries and improvements in the medical and surgical sciences. Edited by Hobart Emory Hare, M. D., Professor of Therapeutics, Materia Medica and Diagnosis in the Jefferson Medical College, Philadelphia; assisted by Leighton F. Appleman, M. D., Instructor in Therapeutics, Jefferson Medical College, Philadelphia. Vol. XXI. No. 1. March 1, 1918. $6.00 per annum. Philadelphia, Lea & Febiger.

This quarterly installment consisting of 307 pages deals with the surgery of the head, neck and breast, and thorax. One article is devoted to infectious diseases, including acute rheumatism, croupous pneumonia, and influenza. Another article reviews the diseases of children, and the last contribution covers rhinology, laryngology and otology.

---

**The Medical Clinics of North America.** Volume 1 Number 4 (The Boston Number, January 1918). Octavo of 401 pages, 128 illustrations. Philadelphia and London: W. B. Saunders Company, 1918. Published bi-monthly. Price per year: Paper $10.00; Cloth, $14.00

This installment contains 18 articles, contributed by 20 authors. There is a wide range of subjects, and they are treated in a liberal style, making free use of illustrations, diagrams and radiographs. Number 4 fulfils the expectations raised by the great success of the first three numbers of the Clinics.

---

**A Manual of Histology.** By Henry Erdmann Radasch, M.Sc., M.D., Assistant Professor of Histology and Embryology in the Jefferson Medical College. Cloth. Price $2.50 net. Pp. 580, with 307 illustrations. Philadelphia: P. Blakiston's Son & Co., 1918.

We have here a clear and readable exposition of normal human histology, which serves a better purpose than the author's well known quiz compend and serves it acceptably. It is handy alike for the student and the practitioner; lucidly written and clearly illustrated; combining in a useful way the main outlines of embryology with the histology of tissues and organs. Frequent references to function serve at the same time to make more complete the descriptions of structure.

---

**A Text-Book of Obstetrics.** By Barton Cooke Hirst, M. D., Professor of Obstetrics in the University of Pennsylvania. Eighth edition, revised and reset. Octavo of 863 pages, with 715 illustrations, 38 of them in colors. Philadelphia and London: W. B. Saunders Company, 1918. Cloth, $500 net.

During the twenty years that this book has been in the hands of the medical profession it has become justly famous for its clear exposition and excellent illustrations. The eighth edition merits the favor that has always been awarded to its predecessors. As a revision, the book is rather unique in that the author has condensed the text and omitted some unessential material in order "to present a concise statement of the best means at our command at present dealing with the numerous complications and consequences of the process of generation." The result is a volume of some 150 pages less than the seventh edition—a shortening accomplished in part by the discarding of more than one hundred illustrations. The price remains the same, however, for which favor many students will be grateful during these times of war prices. The reader who is disappointed by the slight attention given to some topics, such as "twilight sleep" for instance, may compensate himself with the assurance that nothing really essential is overlooked in this generous volume. It will easily maintain its rank as one of our most reliable authorities. The publishers have done their part of the work handsomely and substantially.

